# Formants provide honest acoustic cues to body size in American alligators

**DOI:** 10.1038/s41598-017-01948-1

**Published:** 2017-05-12

**Authors:** Stephan A. Reber, Judith Janisch, Kevin Torregrosa, Jim Darlington, Kent A. Vliet, W. Tecumseh Fitch

**Affiliations:** 1 Department of Cognitive Biology, University of Vienna, Vienna, 1090 Austria; 2 St. Augustine Alligator Farm Zoological Park, St. Augustine, FL 32080 USA; 3 Wildlife Conservation Society, Bronx Zoo, Bronx, NY 10460 USA; 4 Department of Biology, University of Florida, Gainesville, FL 32611 USA

## Abstract

In many vertebrates, acoustic cues to body size are encoded in resonance frequencies of the vocal tract (“formants”), rather than in the rate of tissue vibration in the sound source (“pitch”). Anatomical constraints on the vocal tract’s size render formants honest cues to size in many bird and mammal species, but it is not clear whether this correlation evolved convergently in these two clades, or whether it is widespread among amniotes (mammals, birds, and non-avian reptiles). We investigated the potential for honest acoustic cues in the bellows of adult American alligators and found that formant spacing provided highly reliable cues to body size, while presumed correlates of the source signal did not. These findings held true for both sexes and for all bellows whether produced in or out of water. Because birds and crocodilians are the last extant Archosaurians and share common ancestry with all extinct dinosaurs, our findings support the hypothesis that dinosaurs used formants as cues to body size. The description of formants as honest signals in a non-avian reptile combined with previous evidence from birds and mammals strongly suggests that the principle of honest signalling via vocal tract resonances may be a broadly shared trait among amniotes.

## Introduction

Acoustic cues in animal vocal signals can provide receivers with a host of biologically relevant information^1^. Advertisement calls may convey a producer’s attributes such as competitive abilities^2^, hormonal state^3^, or body size^4^. Such vocalisations may be under strong selective pressure to be “honest signals”, as dishonest signals would eventually be ignored by receivers, rendering the signal ineffective^5, 6^. The evolutionary mechanisms ensuring signal honesty remain a topic of debate. The “handicap principle”^7^, the idea that a signal can be kept honest by being costly, has received theoretical support^8^, but has been challenged theoretically^9–11^ and empirically^12^ by later authors. In stable groups with repeated interactions, low cost honesty can be enforced by a high cost of discovered dishonesty^13^, and honest signals may be cheap when the signaller and the receiver share kinship^14, 15^. Another possibility is that physical constraints on signal production can enforce honesty directly^16, 17^. For example, the body weight of funnel-web building spiders (*Agelenopsis aperta*) determines their web vibrations, so that a conspecific receiver can reliably predict a potential opponent’s competitive abilities^18^.

In the particular case of advertisement calls in amniotes (mammals, birds, and non-avian reptiles), the honesty of acoustic signals may be maintained via anatomical constraints on the vocal apparatus^19–21^. Constraints could theoretically act on either of the independent contributors of the vocal sound production system in amniotes: the source or the filter^21, 22^. Vibrating tissue, e.g. the vocal folds in the mammalian larynx, produces the source signal, which is subsequently filtered in the vocal tract with certain frequencies being amplified and others attenuated^23, 24^. Early research suggested that the rate of tissue vibration in the source (the fundamental frequency, ƒ0) could represent a reliable cue to a caller’s body size^25^. However, it was empirically determined that in many species there is no apparent link between fundamental frequency and body size^26–29^, possibly because an animal’s anatomy puts few restrictions on the size of vibrating tissues in the sound source^30^. Filter properties, on the other hand, are strongly constrained by anatomy, and the vocal tract length often shows a strong positive correlation with overall body size within a given species^19, 30^. It follows that the resonance frequencies of the vocal tract, termed “formants”, often inversely correlate with vocal tract length and hence that larger individuals produce calls with lower resonance frequencies.

Formants have been shown to be reliable cues to a caller’s body size in multiple mammalian taxa^21, 31, 32^ and bird species^33–37^. In mammals, supra-laryngeal cavities form the vocal tract. In birds the source signal is produced in the syrinx, an organ at the base of the trachea, and the vocal tract additionally includes the trachea as well as supra-laryngeal cavities. For both taxonomic groups, mammals and birds, adaptations to exaggerate vocal tract length have also been described with some mammals lowering the larynx permanently^38^ and/or during vocalisation^20, 39^ and some birds developing an elongated trachea^33^. But because such anatomical constraints may remain the same for all individuals within a species, formants can nonetheless remain honest cues to body size even in such cases^4, 19^. These examples illustrate that selection can act on the filter to produce lower formants, and that the same principle of honest signalling occurs in two phylogenetically distant taxonomic groups. Interestingly, formants are not known to play an active role in amphibian vocalizations^40^. So an important open question is whether honest signalling via formants represents a convergently evolved trait in birds and mammals or whether it represents a basal property of amniotes.

To date, no honest acoustic cues have been described in adult non-avian reptiles (hereafter “reptiles”), and most species in two of these clades (Squamata - lizards, Chelonian - turtles) show little vocal behaviour (with exceptions, e.g. geckos) directed towards conspecifics^41^. Extant crocodilian species in contrast frequently produce vocalizations in multiple contexts^42^ and are thus excellent candidates to investigate whether formants represent honest acoustic cues to body size. Recent studies have shown that vocal communication plays an important role in crocodilian social ecology^43^. Pre-hatching calls synchronize hatching time and alert mothers to incipient hatching^44^; juvenile vocalisations grade from contact into distress calls and receivers respond accordingly^45^. In juvenile Nile crocodiles (*Crocodylus niloticus*), the fundamental frequency of distress calls scales with age and adult females differentiate between the calls of larger and smaller juveniles^46^.

Relative size differences are also important following sexual maturity, with females only accepting males larger than themselves as mates^47^ and larger individuals reliably prevailing in agonistic interactions^48, 49^. Because both sexes compete for access to breeding areas and mates^50^, honest acoustic cues to body size in mature animals would provide highly relevant information in both sexes.

The most conspicuous vocal behaviour of adult crocodilians is bellowing and roaring: loud, low-frequency long-distance vocalisations produced year-round but most commonly during the mating season^43^. In 1978, Garrick *et al*. suggested in an observational study that bellows of American alligators (*Alligator mississippiensis*) might serve as honest signals of size (*“Moreover, the largest individuals, the males, gave the loudest and deepest bellows and were the most conspicuous during bellowing.”*)^51^. This hypothesis has never been tested, and Garrick *et al*.^51^ did not specify which specific acoustic parameters in the display might convey the size information.

**Figure 1:**
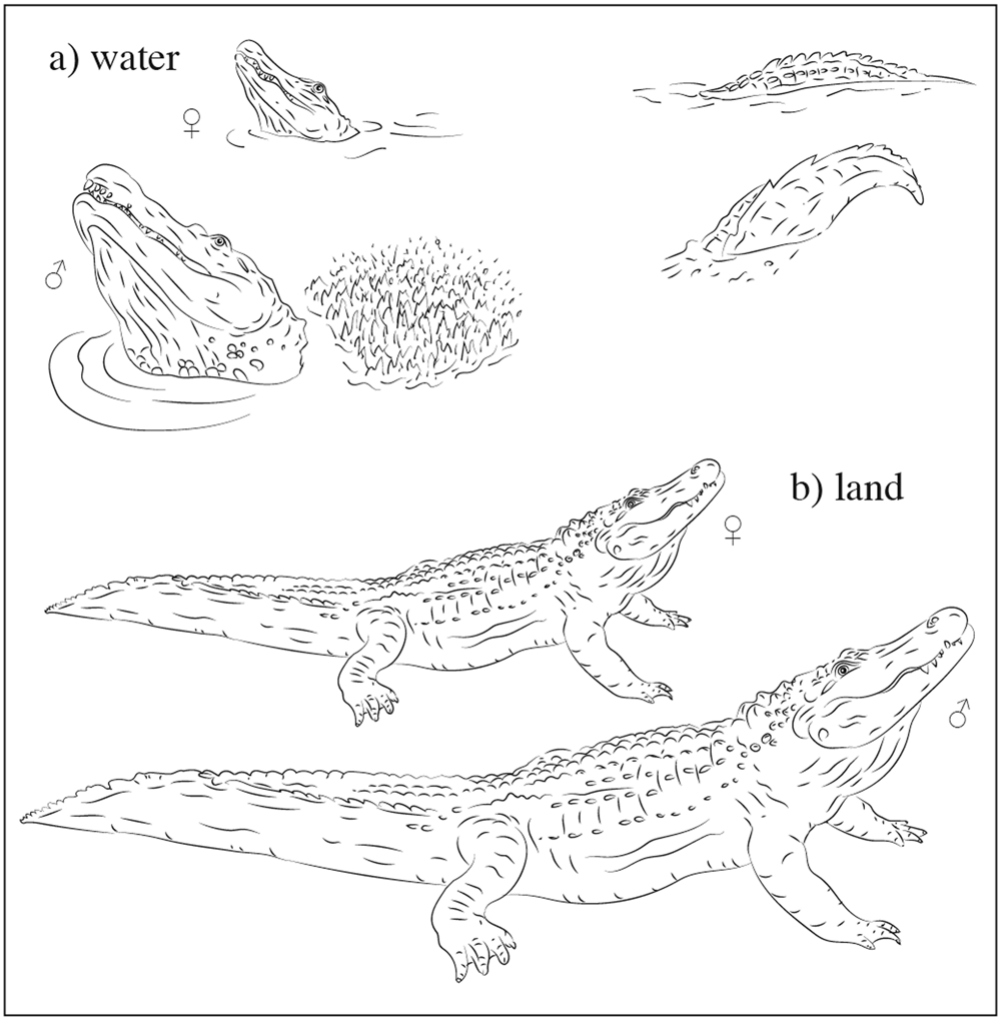
Bellowing displays in American alligators differ between locations and sexes. In water (**a**), only males produce a water dance, where droplets of water are sprayed upward over the thorax, but both sexes raise their head and tail above the surface. On land (**b**) both females and males raise the head but not the tail (drawings by S.R.).

Bellows have several characteristics which make them ideal candidates for advertisement calls. First, bellows are very low in frequency, reducing attenuation over long distances^1^. Second, the bellowing display differs between sexes, at least in the American alligator, in that only males exhibit sub-audible vibrations (visible by the so-called “water dance”, see Fig. 1) before the audible bellow^52^. Finally, bellows encompass a broad frequency range^50^ which would be ideal to outline formants. Thus, crocodilian bellows are good candidate vocalizations to contain honest acoustic cues to body size encoded in resonance frequencies.

In a previous study we provided evidence that bellows of a Chinese alligator (*Alligator sinensis*) female possess formants^53^. When the alligator was breathing heliox (helium-oxygen mixture) instead of ambient air, the formants (visible as high-energy frequency bands) in the spectrum of bellows shifted upwards in frequency, demonstrating that these bands were the result of gas vibrating in the vocal tract. The fundamental frequency could not be measured, as bellows are noisy, chaotic vocalisations with little periodicity. However, we found that the dominant frequency (DF) remained unchanged between the two atmospheres (ambient air vs. heliox) and we concluded that DF represents tissue-generated source vibrations in Chinese alligators.

**Figure 2:**
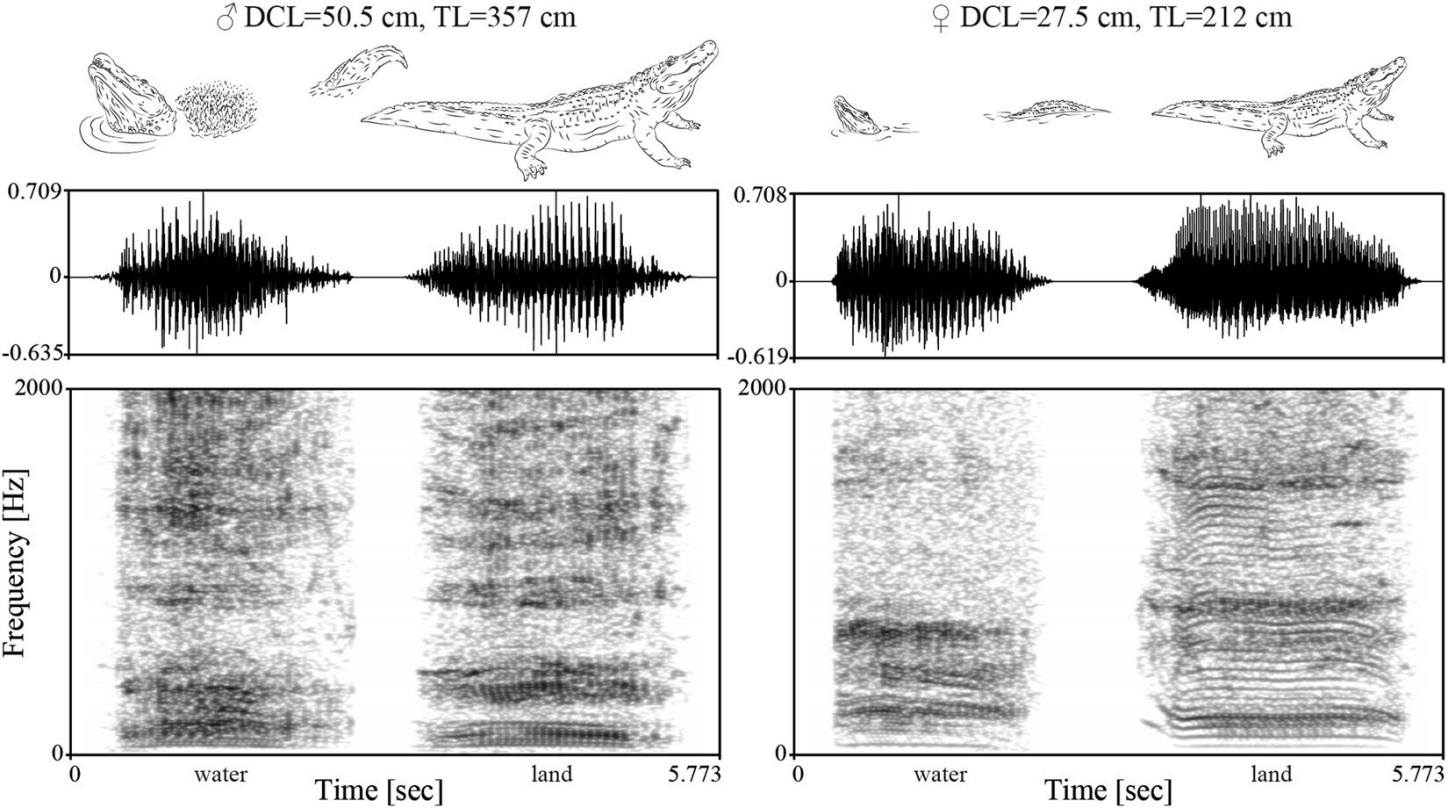
Example recordings of bellows by a male and a female American alligator in water and on land (Peak amplitude equalized to −3 dB in Adobe Audition, Spectrogram settings in Praat: Window length [sec]: 0.12; Dynamic range [rel dB]: 40.0, drawings by S.R.).

In the present study we investigated whether formant frequencies in the bellows of 43 sexually mature American alligators provide cues to the caller’s body size. We measured head length (“dorsal cranial length”^54^, DCL) and total body length (TL) in a group of captive alligators and recorded bellows from each individual. Most subjects bellowed in water (majority of the body submerged, head and tail raised above the water surface, see Fig. 1) and on land (see Fig. 2), and we assigned individual recordings to the production location. We predicted the theoretical frequencies of the first three formants by approximating the subjects’ vocal tracts as half-open uniform tubes of the same length as their DCL. We used the resulting values i) for a semi-automated analysis of the formants in the recordings^53^, and ii) to compare expected and measured formant distribution. We also automatically extracted the dominant frequencies (DF) and manged to measure the fundamental frequency (ƒ0) in about half of the recordings. Using Generalized Linear Mixed Models (GLMM), we investigated whether three acoustic parameters, spacing between the first two formants (F1F2)^31^, DF, and ƒ0 would predict the caller’s body size and additionally controlled for the influence of caller’s sex and production location (water/land). We expected that the formant frequencies would negatively correlate with the body size of the caller, and that dominant and fundamental frequency would only marginally relate to size if at all. To the best of our knowledge, this is the first study addressing the information content of formants in reptiles.

## Results

### Acoustic measurements to body size

The subjects (13 females & 29 males) ranged in DCL from 25 to 57.5 cm (n = 43, mean = 42.5 cm, median = 43.5 cm) and in TL from 197 to 390 cm (n = 37, mean = 313 cm, median = 322 cm). DCL was chosen as the assessment of body size for the subsequent analyses, because i) it could be measured for each recorded subject and ii) DCL and TL were found to be very strongly correlated (Spearman’s rho: n = 37, rho = 0.965, *P* < 0.001, see Supplementary Fig. S1). Although the latter finding was expected, it is noteworthy, because American alligators display allometric growth^55^ and many captive specimens differ in their skull morphology from their wild conspecifics^56^.

The best supported GLMM with F1F2 as the response variable included all three main effects – size (DCL), location (water/land), sex (male/female) - and the two-way interaction between size and sex. All these coefficients significantly influenced the formant spacing. Although the interaction term was significant, additional single-term GLMMs confirmed that F1F2 was an indicator of size in males (*t* = −8.141, *P* < 0.001) as well as in females (*t* = −5.526, *P* < 0.001). The final GLMM for DF also included all main effects, the two-way interaction between size and sex, and additionally an interaction between size and location. Again, all coefficients had a significant effect (for full results see Table 1). Post-hoc single-term GLMMs for the two-way interactions showed that DF was only an indicator of size in females (*t* = −2.983, *P* = 0.009) but not in males (*t* = 0.618, *P* = 0.541), and that DF only related to size for bellows produced on land (*t* = −3.258, *P* = 0.002) and not in water (*t* = −0.306, *P* = 0.761). The final model for ƒ0 included no coefficients besides the random effect.

**Table 1:** Values of the final generalized linear mixed models for F1F2 and DF.

response variable	coefficients	estimate	SE	*t*	*P*	
F1F2	size	−13.254	1.829	−7.245	>0.001	***
	location	−74.873	3.851	−19.441	>0.001	***
	sex	−202.944	76.463	−2.654	0.011	*
	size*sex	4.682	2.078	2.253	0.029	*
DF	size	−7.278	1.564	−4.653	>0.001	***
	location	−69.058	25.434	−2.715	>0.001	***
	sex	−192.565	65.138	−2.956	0.005	**
	size*sex	5.646	1.761	3.206	0.003	**
	size*location	2.116	0.570	3.710	>0.001	***

F1F2 = Spacing between 1^st^ and 2^nd^ formant, DF = Dominant frequency, SE = standard error.

**Figure 3:**
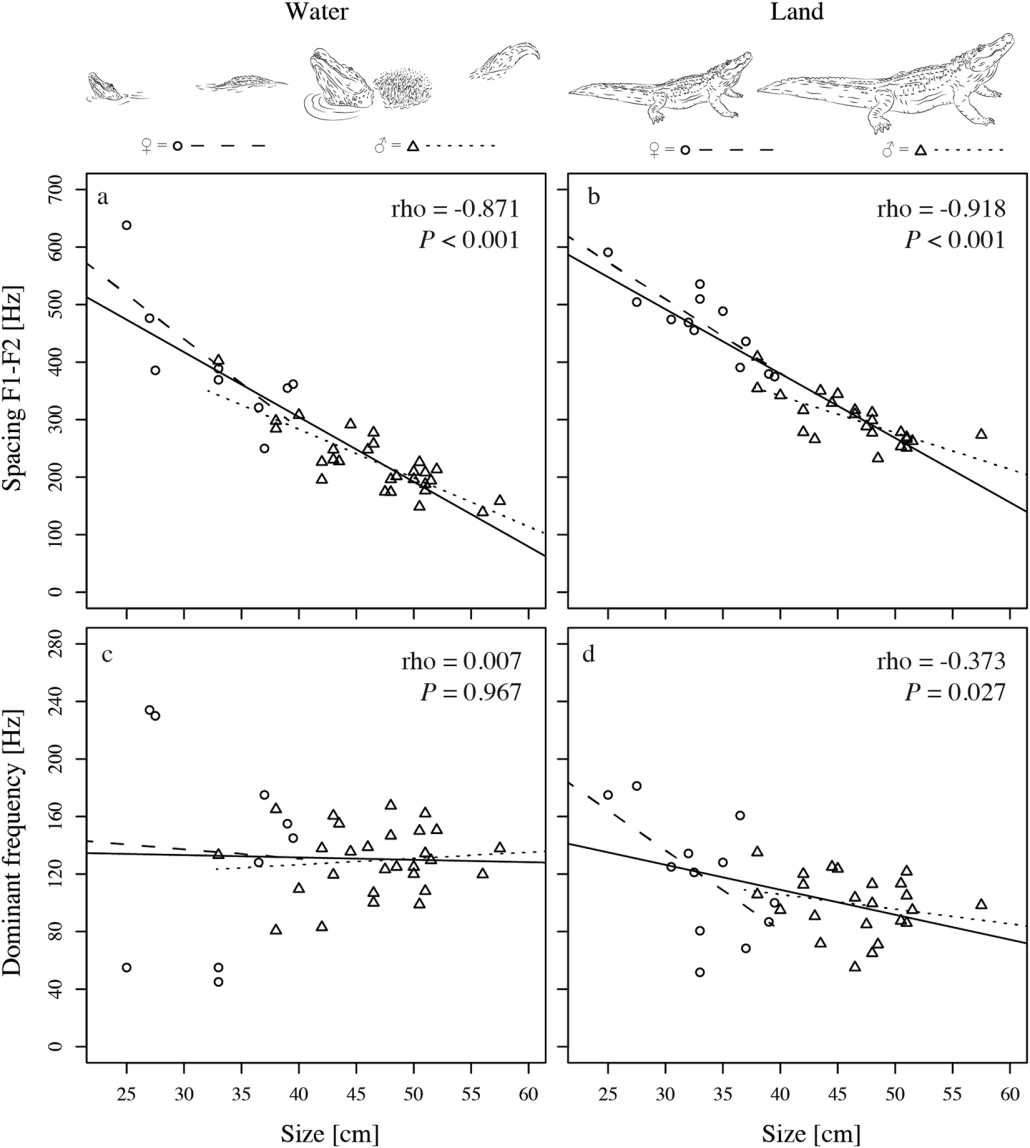
The spacing between the first two formants F1F2 (upper panels) was strongly inversely correlated with size in water (**a**) and on land (**b**) for both sexes. The dominant frequency (lower panels) showed no correlation with size in water (**c**) but correlated weakly for bellows produced on land (**d**). (size represented with DCL, drawings by S.R.).

Formant spacing was strongly, inversely correlated with body size for bellows produced on land and in water over all subjects (Spearman’s rho: rho_min_ = −0.871, rho_max_ = −0.918, *P* < 0.001) and within the sexes (rho: rho_min_ = −0.739, rho_max_ = −0.820, *P* < 0.007, see Table 2 for full results and Fig. 3 for data).

**Table 2:** Spearman’s rho correlation between body size and acoustic variables (mean per individual): coefficients and significance levels.

acoustic variable	subjects	location	n	rho	*P*	
F1F2	all	water	37	−0.871	<0.001	***
		land	35	−0.918	<0.001	***
	females	water	9	−0.820	0.007	**
		land	13	−0.739	0.006	**
	males	water	28	−0.747	<0.001	***
		land	23	−0.755	<0.001	***
DF	all	water	37	0.007	0.967	
		land	35	−0.373	0.027	*
	females	water	9	0.021	0.957	
		land	13	−0.567	0.054	
	males	water	28	0.095	0.632	
		land	23	−0.237	0.275	

F1F2 = Spacing between 1^st^ and 2^nd^ formant, DF = Dominant frequency.

Results were more variable for dominant frequency; DF showed a weak yet significant negative correlation with body size over all subjects for bellows produced on land (rho = −0.377, *P* = 0.027) but not in water (rho = 0.007, *P* = 0.967). There was a statistical trend for a moderate negative correlation between DF and body size for female bellows on land (rho = −0.567, *P* = 0.054) but no correlations were found for males, or for calls uttered in water (rho_max_ = −0.237, *P* > 0.275, see Table 2 for full results and Fig. 3 for data).

The fundamental frequency showed no correlation with size (rho = −0.03, *P* = 0.847, for data see Supplementary Fig. S2).

### The effect of bellowing location on formants and vocal tract shape

The location in which bellows were produced had significant effects on formant frequencies: The first formant was consistently lower in bellows produced on land than in water (exact Wilcoxon signed-rank test, n = 29, *Z* = 3.298, *P* < 0.001, see Fig. 2 for spectrograms). When compared with the expected value for F1 (obtained with equation (1), see Methods), based on the assumption that an alligator’s DCL approximates its vocal tract length, the first formant measured was on average 4.8% higher than predicted if the bellow was produced in water (n = 37, *Z* = −2.738, *P* = 0.005) and 8.1% lower if the animal bellowed on land (n = 35, *Z* = 3.374, *P* < 0.001). However, the second formant (measured) was found to be higher in bellows on land than in water (n = 29, *Z* = −3.925, *P* < 0.001), indicating that the shape of the vocal tract was affected by the bellowing location.

**Figure 4:**
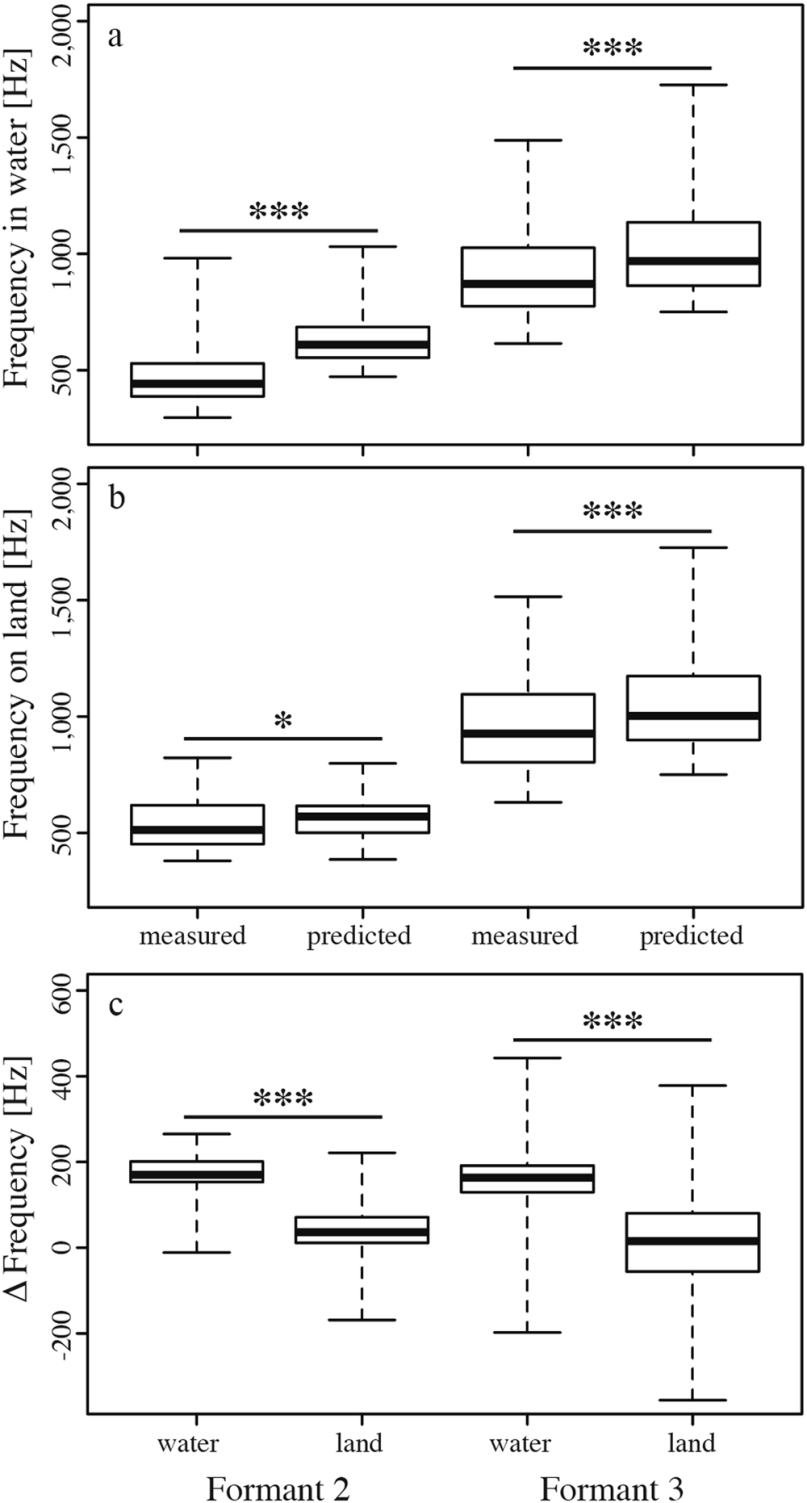
The theoretically expected frequencies of F2 and F3, calculated based on measured F1, were higher than the measured F2 and F3 both in water (**a**) and on land (**b**). The differences between the measured and the predicted formants (∆ Frequency) were lower on land than in water for both F2 and F3 (**c**) (*P ≤ 0.05; **P ≤ 0.01; ***P ≤ 0.001), indicating that vocal tract configuration is closer to a uniform tube during land-based bellows.

The shape of the alligators’ vocal tracts diverged less strongly from the theoretical half-open uniform tube for bellows produced on land. The second (F2) and the third (F3) formant were lower than expected based on calculation with the first formant (F1) (employed in equation (1)). The calculated values compared with the measured values for F2 and F3 in the following manner: For bellows produced in water, F2 was on average 26.1% (n = 37, *Z* = −5.273, *P* < 0.001) and F3 9.6% (n = 37, *Z* = −5.303, *P* < 0.001) lower than expected (Fig. 4a); for calls on land F2 was lower by only 4.7% (n = 35, *Z* = −2.408, *P* = 0.015) and F3 by 10% (n = 35, *Z* = −5.159, *P* < 0.001, Fig. 4b). Overall, the difference between the theoretical position of the formants, calculated by approximating the vocal tract as a uniform tube, and the measured formants from the recordings was smaller on land than in water for both F2 (n = 29, *Z* = 4.379, *P* < 0.001) and F3 (n = 29, *Z* = 3.233, *P* < 0.001, Fig. 4c). These results indicate changes in vocal tract configuration between land and water.

## Discussion

We found that formants in bellows of the American alligators analysed here provide highly accurate acoustic cues to body size, and that the spacing between the lowest two vocal tract resonances is a reliable indexical cue to body size in both males and females. This was true for bellows produced on land and in water, although we also observed differences in acoustic structure between these two production locations. The dominant frequency, presumed to be related to source vibrations, on the other hand is a very poor indicator; we only found a weak link between body size and DF for bellows produced on land, but none in water. The fundamental frequency was not related to size, sex, or production location. Overall, our findings are consistent with the hypothesis that anatomical constraints enforce honesty^17, 19^ in vocal advertisement in crocodilians, and that the “honest” cues are encoded in formants rather than source parameters.

We previously predicted that the vocal tract during bellowing consists of the nasal and the pharyngeal cavity sealed off from the oral opening with the palatal valve pressed against the palatal fold^53^. Because larger animals with longer heads have longer nasal and more spacious pharyngeal cavities, we hypothesized that the frequency of the vocal tract resonances would inversely correlate with vocal tract length and hence with body size. Our results strongly support these predictions and suggest that bellows of American alligators, and perhaps crocodilians in general, act as “honest” advertisement calls conveying size, as has previously been hypothesized^51^.

In contrast to the vocal tract resonances, presumed source vibrations (DF, ƒ0) contain only weak or non-existent cues to size. The dominant frequency in the bellows of a Chinese alligator was not affected by a change in atmosphere between ambient air and heliox^53^, which suggests that in this species, DF is a component of the source signal. Because the Chinese alligator is the closest living relative of the American alligator we presume, but cannot know with certainty, that DF in American alligator bellows is caused by tissue vibration as well. It remains unclear whether the vibrating source tissue is the vocal folds. The small crocodilian vocal folds are not highly specialized and their length essentially equals the diameter of the trachea. Unlike the mammalian epiglottis the palatal valve does not protect the trachea during swallowing food but prevents water from flowing into the pharyngeal cavity and through the glottis. The larynx’s main function might be to seal the trachea during swallowing and can hence not accommodate specialized vibrating structures, which could also block the immediate access to respiratory air or become injured by largely unprocessed pieces of prey. Thus it remains unclear if source vibrations relate to the vocal folds or another vibrating tissue.

Many of our study animals bellowed on land as well as in water, allowing us to compare bellows of the two locations within subjects. Although the formant spacing F1F2 was a strong predictor of body size in either location, the formant distribution differed: The first formant was consistently lower, and the second one higher, on land. The formant distribution indicated that the configuration of the vocal tract resembled a uniform tube more closely in bellows produced on land. As mentioned above, the caudal section of the vocal tract consists of the pharynx, and this is probably affected by submergence in water, in contrast to the rostral section with the bony nasal cavity. How exactly the vocal tract configuration changes due to the different circumstances will probably only become clear once cineradiographic recordings can be obtained from a bellowing alligator. One hypothetical explanation would be that alligators actively extend their vocal tract by lowering their larynx during bellowing. They might retract their larynx, and thereby the massive tongue, towards their sternum with sterno-hyoid muscles, pressing the palatal valve firmly against the palatal fold in the process. In this position the vocal tract, consisting of nasal and pharyngeal cavity, would be maximally extended in length. If this were the case, the behaviour would probably be more readily performed on land, since the lower F1 suggests that the vocal tract is longer and more stretched on land. Water might put more pressure against the pharyngeal area or its buoyancy could act on the pouch containing the tongue. All crocodilians raise their head during bellowing but in water only, they must also lift the tail above the surface to remain in a stable position (“head oblique tail arched” posture)^52^. The two different postures or the difference in strain to maintain them might also affect the configuration of the vocal tract.

Although sex was a significant predictor of bellow acoustics in generalized linear mixed models looking at formant spacing and DF across all subjects, within-sex analyses showed that both sexes followed the overall pattern of a strong inverse correlation of body size with formant spacing. Given that sex and size are highly related in our study species, a biologically-meaningful sexual dimorphism in formant spacing independent of body size is unlikely. American alligators display a strong sexual dimorphism in body size with females hardly ever reaching, and most males exceeding, three meters in TL. In our study group, only one male was shorter than the three longest females and only three males had about the same DCL as the largest females (within < 2 cm). Hence sex and size are strongly related and the two-way interaction between these coefficients indeed significantly affected all of our models with the exception of the GLMM looking at ƒ0. Additionally, we had an uneven sample size between the sexes in our study group. For an accurate sex comparison, we would need a more balanced number of subjects of both sexes and of the same size distribution.

The animals in our sample were all sexually mature; but like most crocodilians, American alligators of either sex start to bellow long before reaching reproductive age and continue to grow thereafter^57^. Alligators might hence not be able to perceive the sex of a caller just based on the bellows’ formant spacing, especially in wild populations with all age groups present and bellowing. However, as already mentioned in the introduction, only males produce a water-dance before a bellow^52^. Alligators have integumentary sensory organs all around their exterior mouth region^58^, which could detect water-borne pressure changes such as the vibrations of a water-dance^47^. A bellowing display could hence function as a two-part signal, the first part advertising sex and the second body size. A receiver might at first perceive whether an out-of-sight bellower is of the same or the opposite sex based on the presence or absence of a water-dance component. Listening to the second/vocal part of the bellowing display, the receiver could then assess the caller’s body size by the formant spacing, and thereby evaluate the potential competitor’s or mate’s quality and resource-holding potential.

At present, almost nothing is known about the perception of adult crocodilian vocalisations. Multiple playback studies, using paradigms previously employed in birds and mammals will be required to clarify whether American alligators can perceive the acoustic cues to body size^59, 60^, and whether they respond differently to a bellow depending on their own sex^31, 61^ and the sex of the caller. The latter point would be of particular interest, because both sexes are equally vocally active in American alligators, unlike many of the better-studied mammalian species.

Honest acoustic signals via formants have presently been demonstrated in both extant archosaurian clades: in birds and now in at least one species of crocodilian. These taxonomic groups share a common ancestor with all extinct dinosaurs^62^. Some dinosaur species, such as Lambeosaurines, had bony crests on their skulls: hollow extensions of their nasal cavity (e.g. *Parasaurolophus walkeri* nasal cavity length: 346 cm)^63^. It has been suggested that these structures acted as cavity resonators functionally analogous to the tracheal elongations found in some recent birds^33, 64^. Our findings support the hypotheses that formants might have played an important role in the communication of extinct Archosaurians.

The principle rendering advertisement calls honest signals by anatomical constraints on the size of the vocal tract has now been described in mammals, birds, and a reptile. The vocal anatomy of these taxonomic clades includes source structures which are not homologous among amniotes, such as the bird syrinx. But in each clade it has now been demonstrated that formants provide honest cues to a caller’s body size. This suggests that formant-based indicators of size did not evolve convergently in birds and mammals, but represent an ancient ancestral constraint on advertisement call evolution throughout the amniotes.

## Methods

### Study animals and location

All data were collected at the St. Augustine Alligator Farm Zoological Park located in Saint Augustine, Florida, USA between March and June 2013. The subjects were 43 sexually mature American alligators. Most of the animals (13 females and 29 males) were kept together in an outdoor enclosure (roughly 557 m^2^) with ponds, an island, and several sand beaches (two adult albino alligators, a male and a female, were singly housed in roofed enclosures for protection against sunburn). The two largest males in the group were wild caught; all other animals were hatched and raised in captivity. All alligators were accustomed to daily interactions with their keepers and roughly half would not retreat when approached and touched. Each study alligator had an implanted microchip under its nuchal plates for identification. In addition, the animals could be individually discriminated by specific characteristics such as tooth position, scars, head shape, coloration, osteoderm and scale patterns. Previous to data recording, a profile of each subject was created. Its chip was read and its particularities photographed from different angles (Canon Powershot SX50).

### Measuring head and total length

Dorsal cranial length (DCL) was measured with two different methods depending on how comfortable a given animal was with humans. Alligators trained for human handling (~25% of the subjects) tolerated a steel measuring tape being placed on their head. For untrained animals, a custom measuring pole was crafted by orthogonally connecting two polyvinylchloride (PVC) pipes; one of the pipes was subsequently marked in one-cm-steps starting from the joint, the other was used as a handle. The alligators were approached while resting on land. The measuring pole was slowly lowered on their head with the rim of the joint being placed behind the caudal edge of the cranial platform. A second person stood alongside the animal and photographed the head and measuring pole. Later, using the image, DCL was assessed along a line orthogonal to the measuring pole from the tip of the rostral end of the skull to the cm markings (see Supplementary Fig. S3).

The subjects’ total body length (“total length”, TL) from nose to tail tip was measured with a novel method. When a given alligator was on land, one researcher (S.R.) approached it from behind and placed a laser-distance-measurement device (BOSCH DLR130 Distance Measurer) mounted on a small tripod at its tail tip aligning the laser source with the posterior tip of the tail. If necessary, the tail was carefully pulled towards the tripod to straighten it out. Then a second researcher (K.T. or J.D.) placed a bamboo stick in front of the alligator’s nose. If the animal was lying straight, the bamboo touched the tip of the nose; if the head was placed at an angle, the second person estimated the distance missing and positioned the stick accordingly. The laser beam was directed at the stick and the resulting distance recorded (Supplementary Fig. S4). Six individuals were too shy to be approached on land (resulting in n = 37 for TL, see Supplementary Material for an empirical evaluation of this novel methodology, including Supplementary Fig. S5).

### Sound recording

All bellows analysed in this study were spontaneously emitted; none were stimulated by playbacks. Between March and May 2013 bellows of the subjects were recorded *ad libitum* every day between 7:30 and 11:00. During the data collection period most study alligators bellowed frequently, some almost daily. However, in alligators bellowing often elicits chorusing and most of the time several individuals were vocalizing simultaneously. Special care was taken to obtain bellows from each individual during time windows when it alone was vocalizing. Bellows were recorded with a handheld Sennheiser directional microphone (ME66/K6) connected to a digital sound recorder (Zoom H4n Handy Mobile 4-Track Recorder, at 44.1 kHz sampling frequency and 16-bit amplitude resolution). Simultaneously, the bellowing individual was filmed (Panasonic HC-X909 HD 3MOS Camcorder) with a special focus on body regions enabling individual identification. The data of caller identity were collected by two researchers (S.R., J.J.) and only if two other staff members of the St. Augustine Alligator Farm, very familiar with each animal (K.T., J.D.), after screening the video material, independently confirmed the initial identification of an individual alligator were the corresponding sound recordings used for acoustic analysis.

### Acoustic analysis

Recordings of bellows from identified individuals were extracted and saved using Adobe Audition (version 4.0). Praat (version 5.2.45, www.praat.org) was used to conduct all acoustic analyses. All bellows were first down-sampled (New sampling frequency: 10,000 Hz; Precision: 50 samples) to account for uneven recording distances and to eliminate high frequency background noise (e.g. calls from a nearby parrot aviaries). All files were visually and acoustically checked for interfering background noises and only recordings with a high signal to noise ratio used for the analyses. The final analysis contained 640 calls from 43 alligators (n_*calls/ind*_ = 4–29, mean_*calls/ind*_ = 14.8, median_*calls/ind*_ = 13).

The first three formants (F1-F3) were automatically measured in all recordings in Praat (To Formant ‘burg’ function). In order to find adequate analysis settings, the theoretical positions of the first three formants were predicted based on the physical principles of voice production^24^. Given an approximate average temperature of 23 °C during the recording hours, the speed of sound (*c*) was expected to be approximately 345 m/sec. The DCL measurement for each individual was used as a first crude approximation of the length of the supralaryngal vocal tract consisting of the nasal and pharyngeal cavity. The shape of an alligator’s vocal tract during bellowing was approximated as a uniform tube^19^ that is open at one end (open at the nostrils, closed at the glottis). With these assumed values for the speed of sound and the vocal tract length the predicted frequency of Formant (*i* = 1, 2, 3) could be estimated for each individual using equation (1) (c = speed of sound, *L* = vocal tract length estimate)^24^:

1 $${F}_{i}=\frac{(2i-1)c}{4L}$$

These values were then used to predict the frequency range in which the first three formants would be expected. The automated analysis was standardized by applying the same basic settings (To Formant (Burg method); Max. number of formants: 3; Window length: 0.12 sec; Dynamic range: 40 dB) and by extracting the formants at the time point of maximum intensity within a recording (To intensity; Minimum pitch: 30 Hz). For each individual, the calculated value for the theoretical third formant was rounded up to the next hundred and this value used to set the top of the frequency range for the software (e.g. head length = 51 cm: theoretical position in spectrum for F3 = 846 Hz; Praat: To Formant (Burg method); Max. formant: 900 Hz). Two criteria were employed to evaluate the automatically extracted values: Praat had to be able to i) identify the three formants and to ii) create a visible match between the formant frequencies as indicated in its editing window and the frequency bands of high energy visible in the spectrogram. For the second criterion, each recording was manually checked. In cases where the visual match was not satisfactory, the setting for the top of the frequency range was either reduced or increased by maximally 100 Hz in steps of 50 Hz. Once the optimal analysis parameters had been determined, all recordings from a given individual were analysed using these values only. In addition, the dominant frequency (DF), the frequency with the highest amplitude in the spectrum, was automatically extracted from all recordings (To Ltas; Bandwidth: 10 Hz; Query, Get frequency of maximum; Frequencies: all, Interpolation: None). In order to obtain the fundamental frequency (ƒ0), the calls of the largest (male, DCL = 57.5 cm) and the smallest (female, DCL = 25.0 cm) subject were used to determine settings in Praat capable of automatically measuring the pitch (To Pitch (ac); Max. number of candidates: 15; Silence threshold: 0.35; Voicing threshold: 0.05; Octave cost: 0.05; Octave-jump cost: 0.2; Voiced/unvoiced cost: 0.14; Range: 40–80 Hz). These settings were then applied to each recording. All calls were manually checked for a visible match between the pitch as indicated in Praat’s editing window and the lowest harmonic visible in the spectrogram. Measurements were only considered if i) such a visible match was observed and ii) the presumed harmonic had integer-multiple counterparts. ƒ0 could only be measured in half of the recordings (318 calls, 49.7%), but at least once for every individual (n_*calls/ind*_ = 1–19, mean_*calls/ind*_ = 7.4, median_*calls/ind*_ = 7).

### Statistical analysis

All 640 calls (318 calls for ƒ0) were employed to compute Generalized Linear Mixed Models (GLMM) with the spacing between the lowest two formants (F1F2)^31^, DF, and ƒ0 as the dependent variables. Location (water/land), sex (f/m), body size (DCL), plus the three two-way interactions and the three-way interaction served as the initial coefficients. Subject identity was added as a random effect. The full models were reduced to find the best fit using the Akaike Information Criteria (AIC). Kenward-Roger approximation was employed to obtain degrees of freedom and the t-distribution to get two-tailed p-values^65^. Single-term GLMMs were used for post-hoc analyses of the final models, e.g. in the case of significant interaction terms. For subsequent Spearman’s rho correlation analysis, the mean frequencies were used for each individual.

Finally, deviations from equation (1) above were used as a baseline comparison to gain a better understanding of the shape of the alligator vocal tract during bellowing, and estimate its resemblance to a uniform tube. The measured value for the first formant (F1) was used to calculate the second (F2) and third (F3) formant’s theoretical position and these values were then compared with the measured values for F2 and F3. All pairwise comparisons were conducted with exact Wilcoxon signed-rank tests^66^. Statistical analysis was performed in R (version 3.0.2 GUI 1.62 for Mac, R packages: lme4, lmerTest, pbkrtest, coin).

### Ethics

The procedures performed for this study were all in accordance with the guidelines of the Association for the Study of Animal Behaviour (ASAB) for ethical treatment of animals in behavioural research. The current study was approved by the St. Augustine Alligator Farm research committee in April 2013. All hands-on procedures were part of, or comparable to, the daily keeper-interaction routine of the subjects. Some animals’ body size (DCL & TL) was measured while they were restrained for medical examination unrelated to this study. All sound recordings were obtained opportunistically and were not provoked by artificial stimuli.

## Electronic supplementary material

Supplementary Material
