# Supplementary material for: Formants provide honest acoustic cues to body size in American alligators
Source: Sci Rep. 2017 May 12;7:1816. doi: 10.1038/s41598-017-01948-1 (PMC5431764; doi:10.1038/s41598-017-01948-1)
Supplement: Supplementary file 1 — Supplementary Material [file 41598_2017_1948_MOESM1_ESM.pdf]

Supplementary Material

Formants provide honest acoustic cues to body size in American alligators

Stephan A. Reber, Judith Janisch, Kevin Torregrosa, Jim Darlington, Kent A. Vliet, and W. Tecumseh Fitch

Abbreviations:

DCL= dorsal cranial length, TL= total length, F=formant, DF=dominant frequency, $f0$=fundamental frequency

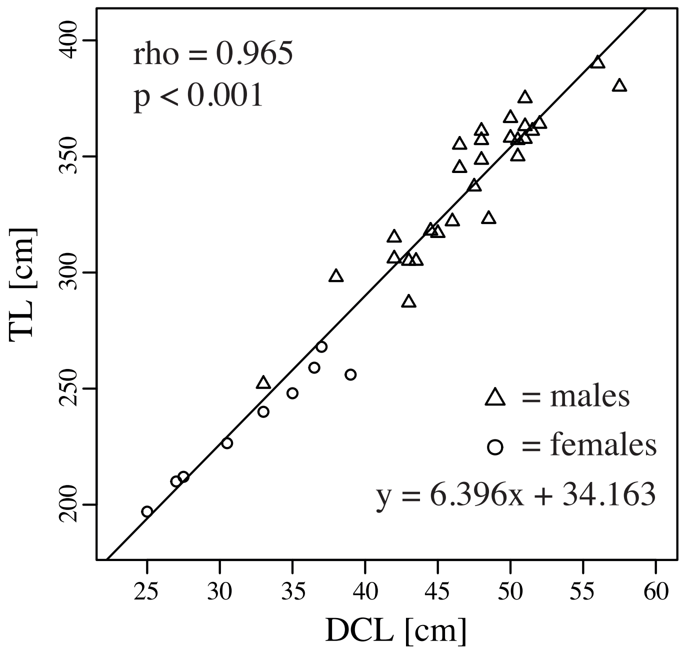

Figure S1. The two measurements of body size, dorsal cranial length (DCL) and total length (TL), were very strongly linearly correlated (Spearman's rho: n=37, rho=0.965, $P<0.001$).

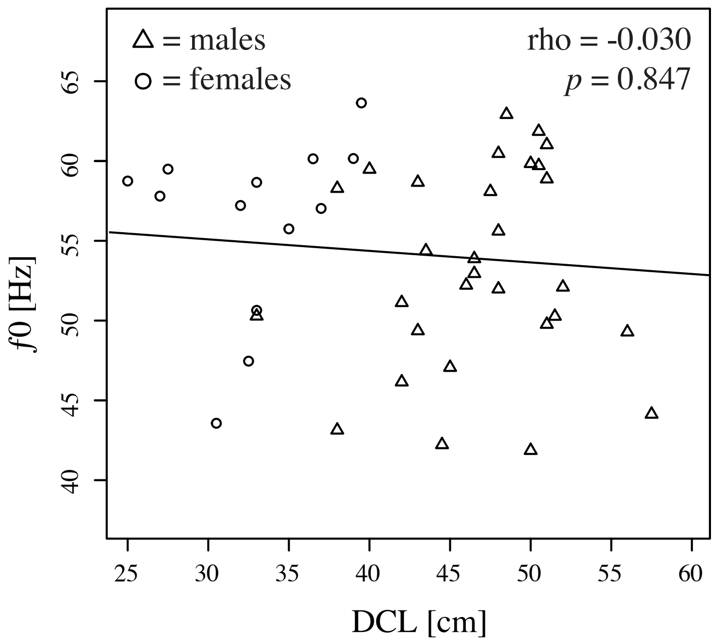

Figure S2. The fundamental frequency of the recorded bellows was not correlated with body size (Spearman's rho: n=43, rho=-0.03, *P*=0.847).

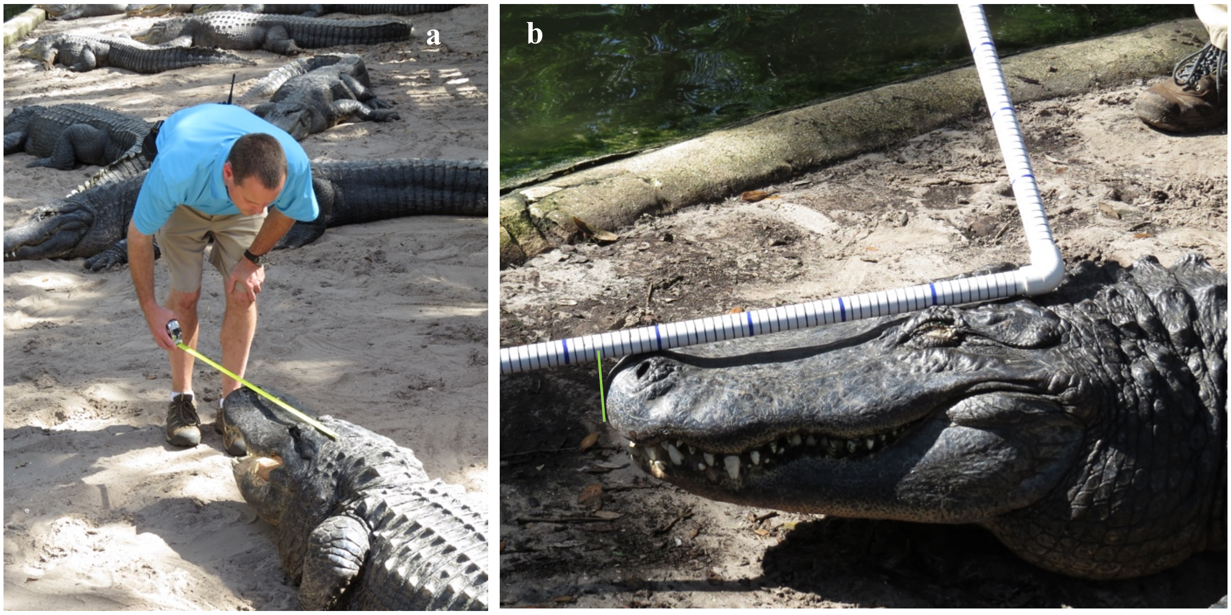

Figure S3. Measuring DCL using a tape measure (a) or a measuring pole (b). After lowering the pole onto the alligator's head, a photograph was taken. DCL was assessed along a line (depicted in light green) orthogonal to the measuring pole from the tip of the rostral end of the skull to the one-cm markings. This male's DCL measures 46.5 cm (photos by S.R.).

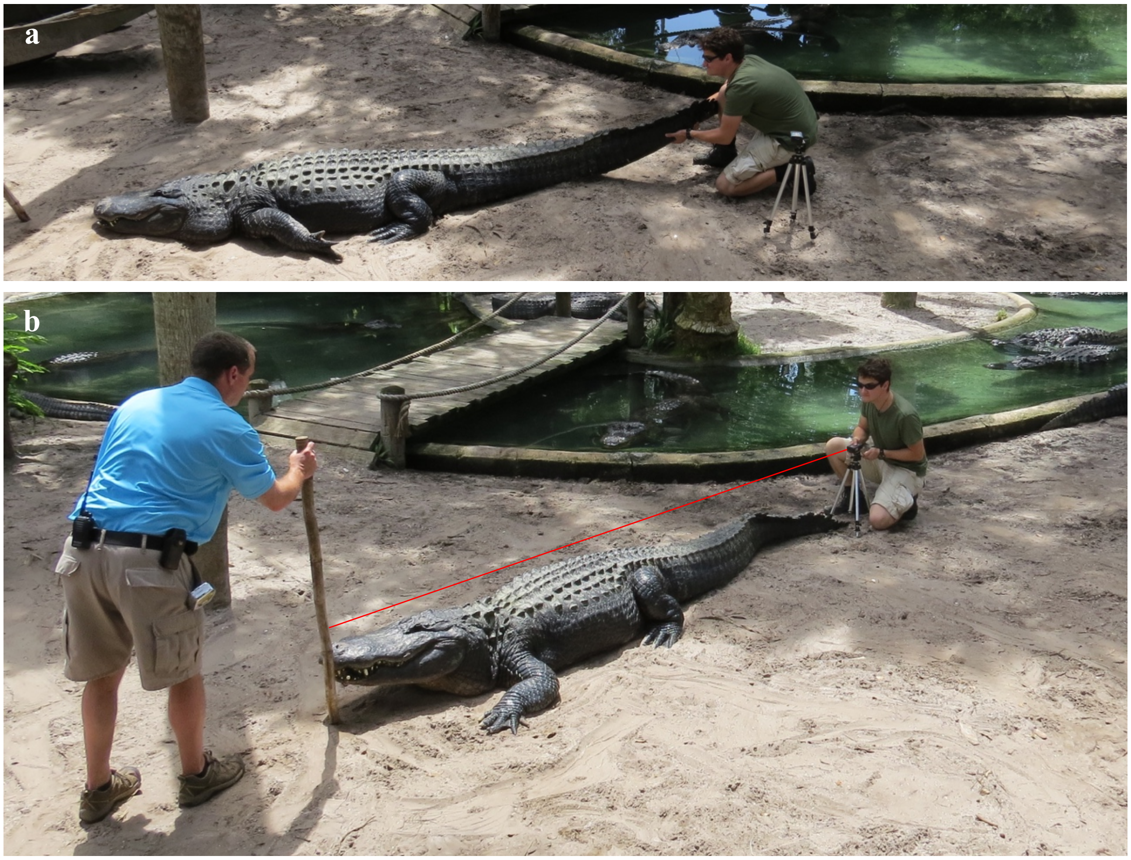

Figure S4. Measuring TL using a laser-distance-measurement device. After pulling the tail straight (a), a researcher placed the laser device at the level of the tail tip and projected the laser beam (depicted in red) onto a bamboo stick held at the nose tip by a second person (b). This male's TL measures 363 cm (photos by J.J.).

Evaluation of the novel length-measurement method using a laser-distance-measurement device

In order to evaluate this new method's reliability, TL of fourteen male alligators was once obtained with the laser-distance-measurement device ("laser method") and a second time using a metal measuring tape ("tape method"). For the latter method - a common approach to measure trained, unrestrained crocodilians in captivity - the tape was extended over the centre back of the alligator, one person held the end at the tail tip, the other calmed the animal in front, lowered the tape onto the head, and read the total length at the tip of the nose. The fourteen males were measured with each method twice by two teams (K.T. & S.R. and J.D. & S.R.). For all measurements the same

person (S.R.) took the position at the tail tip. Assessment of the distance with the laser method or reading the body length at the appropriate moment from the measuring tape was performed by two different researchers (K.T. or J.D.) independently. One measurement was made on an alligator strapped to a transport stretcher (during transport to a novel enclosure), the other thirteen were measured unrestrained in their enclosure. To estimate the inter-measure reliability, the two teams' data sets were compared by calculating the deviations in TL for each animal per method.

The laser method was found to be more reliable than the common approach using a metal measuring tape. The absolute deviations for TL between the two measures were smaller for the laser method (exact Wilcoxon signed-rank test: n=13, Z=-2.125, P=0.032, Figure S5). For the restrained animal, both measures reached close agreement with both methods (tape: 303 cm, laser: 298 cm, 1.65% difference).

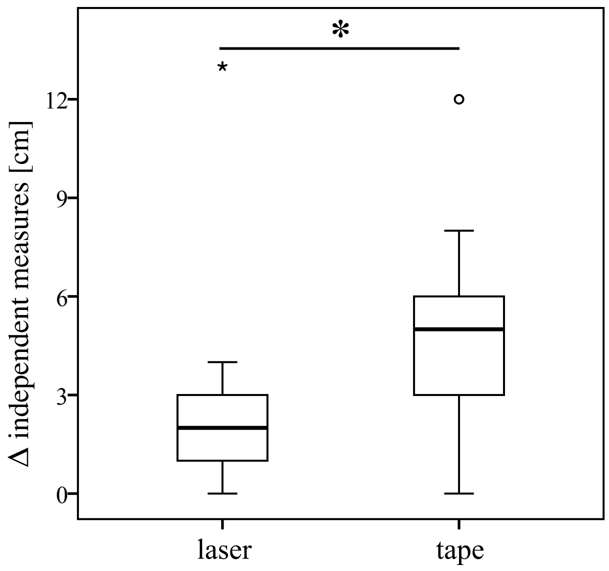

Figure S5. Laser versus Tape measurements: The absolute deviations (Δ) between independent measures are higher for the approach using the measuring tape than for the new method with the laser-distance-measurement device (*P≤0.05).

There are a number of explanations why the tape method created more variable results. A measuring tape can get caught on osteoderms on the animal's back or on tail spikes, causing it to lie unevenly. Trained crocodilians might tolerate a measuring tape on their back, but get excited and start to move in anticipation of a food reward. The laser method was much faster and could under ideal conditions be conducted in a few seconds reducing stress for the animal and risk of injury for the handlers.

Table S1. DCL, TL, mean F1-F3, DF, and $f0$ per subject for bellows produced in water.

subject	sex	DCL [cm]	TL [cm]	F1 [Hz]	F2 [Hz]	F3 [Hz]	DF [Hz]	$f0$ [Hz]
subject_1	m	43.0	287.0	211.454	441.914	614.654	160.556	57.551
subject_2	f	27.5	212.0	305.587	691.256	1348.405	230.000	55.804
subject_3	m	47.5	337.0	190.470	365.018	756.846	123.235	58.084
subject_4	m	38.0	298.0	230.879	528.741	1016.463	165.000	45.901
subject_5	f	39.0	256.0	182.254	537.108	1026.162	155.000	79.415
subject_6	m	51.0	357.5	178.239	366.242	702.107	162.083	61.018
subject_7	m	51.5	361.0	180.764	374.682	703.448	129.615	50.266
subject_8	m	56.0	390.0	157.368	296.388	693.222	119.737	49.284
subject_9	m	43.0	305.0	228.644	476.496	966.594	119.444	49.358
subject_10	m	48.5	323.0	201.256	402.737	793.437	125.000	NA
subject_12	m	46.0	322.0	233.806	481.666	827.757	138.750	52.217
subject_14	m	51.0	375.0	193.455	370.215	783.625	108.158	56.337
subject_15	m	50.5	350.0	172.768	321.360	790.976	150.000	60.341
subject_16	m	44.5	318.0	216.528	507.983	952.845	135.556	43.690
subject_17	m	38.0	NA	215.320	499.671	999.953	80.714	58.158
subject_18	m	50.0	366.5	172.737	382.061	767.764	120.000	59.840
subject_19	m	46.5	355.0	194.493	452.460	834.080	100.000	54.226
subject_20	m	48.0	361.0	199.421	373.445	844.774	167.500	60.471
subject_21	m	50.5	357.0	195.959	422.021	835.998	98.889	62.180
subject_22	m	57.5	380.0	166.811	325.165	670.775	137.857	44.324
subject_23	f	27.0	210.0	295.152	771.470	1488.302	234.091	57.799
subject_24	m	50.0	358.0	203.185	399.574	765.600	125.000	41.859
subject_25	m	52.0	364.0	179.891	393.477	775.090	150.556	52.094
subject_26	m	48.0	348.5	191.260	387.561	826.936	146.667	54.640
subject_27	m	33.0	252.0	227.744	630.339	1166.574	133.182	50.281
subject_28	m	51.0	363.0	184.622	392.819	775.034	134.412	49.762
subject_29	m	40.0	NA	221.070	529.108	913.753	109.583	58.444
subject_30	f	33.0	240.0	272.085	641.317	1189.798	45.000	51.137
subject_31	f	25.0	197.0	343.644	981.625	1478.226	55.000	58.722
subject_33	f	36.5	259.0	268.450	589.570	1127.144	128.077	56.685
subject_35	f	37.0	268.0	257.500	507.420	1102.760	175.000	NA
subject_36	m	42.0	315.0	209.821	405.230	958.132	83.000	51.795
subject_38	f	39.5	NA	175.320	537.011	1074.375	145.000	NA
subject_39	f	33.0	NA	279.332	668.019	1210.174	55.000	60.535
subject_40	m	42.0	306.0	212.192	438.570	974.816	137.857	NA
subject_42	m	46.5	345.0	215.229	492.669	870.785	106.818	NA
subject_43	m	43.5	305.0	192.931	420.569	971.566	155.000	NA

Table S2. DCL, TL, mean F1-F3, DF, and $f0$ per subject for bellows produced on land.

subject	sex	DCL [cm]	TL [cm]	F1 [Hz]	F2 [Hz]	F3 [Hz]	DF [Hz]	$f0$ [Hz]
subject_1	m	43.0	287.0	147.520	413.501	688.800	90.714	59.995
subject_2	f	27.5	212.0	266.154	770.525	1368.330	181.250	60.106
subject_3	m	47.5	337.0	158.646	446.881	741.445	85.000	58.387
subject_4	m	38.0	298.0	186.854	541.212	1020.091	135.000	42.443
subject_5	f	39.0	256.0	199.743	579.062	1054.328	86.739	60.156
subject_6	m	51.0	357.5	128.673	379.661	763.319	121.667	64.433
subject_7	m	51.5	361.0	161.253	423.939	738.602	95.000	51.024
subject_10	m	48.5	323.0	226.926	459.540	757.461	71.000	62.914
subject_11	f	30.5	226.5	179.275	653.329	1176.952	125.000	43.566
subject_13	f	32.5	NA	263.103	718.437	1207.614	121.154	47.458
subject_14	m	51.0	375.0	161.568	431.002	788.885	86.000	60.776
subject_15	m	50.5	350.0	167.787	421.139	782.200	87.500	58.760
subject_16	m	44.5	318.0	192.860	521.805	949.024	125.000	42.223
subject_17	m	38.0	NA	199.288	608.470	1004.201	105.833	58.282
subject_19	m	46.5	355.0	196.520	505.849	851.592	55.000	53.875
subject_20	m	48.0	361.0	179.507	456.703	832.770	65.000	59.427
subject_21	m	50.5	357.0	166.145	444.235	832.578	113.333	61.527
subject_22	m	57.5	380.0	142.371	415.916	631.325	98.333	43.296
subject_26	m	48.0	348.5	159.977	458.675	818.349	113.000	56.568
subject_28	m	51.0	363.0	152.901	419.015	774.746	105.000	52.741
subject_29	m	40.0	NA	245.861	588.125	938.427	95.000	60.925
subject_30	f	33.0	240.0	246.490	756.197	1099.715	80.556	50.641
subject_31	m	45.0	317.0	167.864	512.358	856.572	123.667	47.062
subject_32	f	25.0	197.0	231.834	822.763	1514.973	175.000	58.749
subject_33	f	36.5	259.0	213.345	604.045	1122.385	160.714	60.141
subject_34	f	32.0	NA	206.936	675.909	1223.267	134.333	57.212
subject_35	f	37.0	268.0	190.216	626.264	1092.061	68.333	57.027
subject_36	m	42.0	315.0	202.391	480.588	925.125	112.500	50.457
subject_37	m	48.0	357.0	181.764	493.907	839.978	99.615	51.977
subject_38	f	39.5	NA	191.360	566.145	1071.588	100.000	63.640
subject_39	f	33.0	NA	183.534	719.188	1201.125	51.667	57.726
subject_40	m	42.0	306.0	186.180	502.478	938.243	120.000	46.151
subject_41	f	35.0	248.0	203.911	692.463	1099.344	128.077	55.746
subject_42	m	46.5	345.0	193.781	510.404	868.893	103.571	52.946
subject_43	m	43.5	305.0	260.838	611.006	926.118	71.667	54.367
